# Crude Polysaccharides from Okra Pods (*Abelmoschus esculentus*) Grown in Indonesia Enhance the Immune Response due to Bacterial Infection

**DOI:** 10.1155/2018/8505383

**Published:** 2018-10-09

**Authors:** Sri Puji Astuti Wahyuningsih, Manikya Pramudya, Intan Permata Putri, Dwi Winarni, Nadyatul Ilma Indah Savira, Win Darmanto

**Affiliations:** Department of Biology, Faculty of Science and Technology, Airlangga University, Surabaya 60115, Indonesia

## Abstract

Okra pods were widely consumed by Indonesians to maintain health. The aim of this study was at investigating the potential of crude polysaccharides from okra pods on immune response in mice infected with *Staphylococcus aureus.* Thirty male Balb/C mice were divided into six groups: normal control, negative control, and treatment groups (administration of crude polysaccharides at doses of 25, 50, 75, and 100 mg/kg). Crude polysaccharides were administrated for fourteen days. Furthermore, mice were exposed to *S. aureus* at the fifteenth day. Two weeks after the end of treatment, the parameters were measured. This study showed that crude polysaccharides at a dose of 75 and 100 mg/kg improved phagocytic activity, spleen index, and splenocytes proliferation. Rising of TNF-*α* levels was shown in groups treated with crude polysaccharides at doses of 25, 50, and 100 mg/kg. All treatment groups showed a decreasing level of IL-17. Crude okra polysaccharides also showed a slight increase in NK cells activity and IFN-*γ* level. Thus, crude okra polysaccharides could act as an effective material to enhance immune response including phagocytic activity, spleen index, splenocytes proliferation, and control immune responses through cytokine production.

## 1. Introduction

The human body is surrounded by environment-contained microbes, including extracellular bacteria, *S. aureus.* These bacteria are able to cause nosocomial infection which can result in serious infections [1]. Normally, immune-related cells will inhibit *S. aureus* transmission but the bacteria also release components against the immune system. Therefore, the body needs a particular compound to enhance the immune response.

Dietary phytochemicals from plants may play important roles in the prevention of many diseases [2]. Plant polysaccharides have been known as an important immunostimulatory agent with broad spectrum, low toxicity, and few side effects [3]. If polysaccharides are component of our daily food, it will give many health benefits for human body.

Okra (*A. esculentus*) is vegetable crop used as food and traditional medicine for many diseases such as dysentery and diarrhea [4, 5]. Okra contains flavonoid and vitamin C as antioxidant and polysaccharides as an immunomodulator [6, 7]. A study with cyclophosphamide as an antigen has revealed that okra polysaccharides increased spleen index, splenocyte proliferation, and cytokines secretion [7]. The extract of okra increased IL-12 secretion and decreased IL-10 secretion in dendritic cells [8]. Related with bacterial infection, okra fruit has high tannins that could abolish bacteria [9].

However, studies have not been reported on the potential of crude polysaccharides from okra pods consumed in Indonesia to overcome high cases of *S. aureus* infection. To further investigate the potential of crude okra polysaccharides, the present study explored the effect by evaluating phagocytic activity, cytokine production, spleen index, splenocytes proliferation, and NK cell activity.

## 2. Materials and Methods

### 2.1. Materials and Chemicals

Okra pods were collected from the traditional market in Surabaya, Indonesia, in May 2017. The okra pods were packaged 500 g per polyethylene bag and then stored at −20°C until use. *S. aureus* (ATCC 25923) was purchased from Balai Besar Laboratorium Kesehatan, Surabaya, Indonesia. RPMI-1640 was purchased from Gibco (Invitrogen Co, Massachusetts, USA). Lipopolysaccharide (LPS) from *Escherichia coli* 055 : B5, L2880, and lyophilized powder were purchased from Listlab (List Biological Labs, Inc., California, USA). 3-(4, 5-dimethylthiazol-2-yl)-2, 5-diphenyltetrazolium bromide (MTT) and human hepatoma cell line (huh7it) were acquired from Institute of Tropical Disease, Airlangga University (Surabaya, Indonesia). Interleukin-17 (IL-17), interleukin-23 (IL-23), interferon-*γ* (IFN-*γ*), and tumor necrosis factor (TNF)-*α*, enzyme-linked immunosorbent assay (ELISA) kit were purchased from BioLegend (BioLegend, Inc., San Diego, USA). All other chemicals and solvent used were of analytical reagent grade.

### 2.2. Preparation of Crude Polysaccharides from Okra Pods

According to Ramesh et al. [29] and Chen et al. [7], frozen okra pods (500 g) were cleaned with distilled water, cut into small slices, homogenized, and macerated with 500 ml double-distilled water (ddH2O) overnight. The extract of okra was filtered and macerated twice again with 300 ml ddH2O. The supernatants were collected by centrifugation at 4300 rpm for 5 min. The supernatants were precipitated by the addition of anhydrous ethanol 1X sample volume and incubated for 24 h at 4°C and then centrifuged again as above. The precipitated material was then dissolved in ddH_2_O and dialyzed through cellulose membrane (Sigma-Aldrich, retaining > Mw 14,000) for 24 h. The aqueous solution was then collected from the dialysis bag and freeze-dried to obtain the crude okra polysaccharides.

### 2.3. Determination of Polysaccharides Content in Okra Pods

Polysaccharides content in okra pods was determined using phenol sulphuric acid assay. Sample solution of crude okra polysaccharides was made from stock of crude okra polysaccharides (10 *µ*L) and aquadest (90 *µ*L). Then, 50 *µ*L of phenol 5% was added. After being homogenized for 1 min, 2 mL of sulphuric acid was added to the solution and incubated for 10 min in room temperature. The blank solution was made from 50 *µ*L of phenol 5% and 100 *µ*L of aquadest. The absorbance was measured at 490 nm.

### 2.4. Animals

Male BALB/c mice (8–10 weeks old, 30–40 g) were provided by the Laboratory Animal from Faculty of Pharmacy, Airlangga University, Surabaya, Indonesia. The animals were maintained in cages made of a plastic with a lid made of woven wire cage at 20°C, with 12 h light/12 h dark cycle, fed and watered by ad libitum. All procedures involving animal care were approved by Animal Care and Use Committee (ACUC) of Veterinary Faculty, Airlangga University, Surabaya, Indonesia, no. 714-KE.

### 2.5. Experimental Design

After 10 days of acclimatization, BALB/c mice were randomly divided into six groups (KN: normal control without any treatment; K−: negative control exposed by *S. aureus* without okra crude polysaccharides administration; P1, P2, P3: okra crude polysaccharides doses 25, 50, 75, and 100 mg/kg BW, respectively). Okra crude polysaccharides were administrated by gavage in fourteen days. Furthermore, mice were exposed to *S. aureus* (0.5 Mc. Farland) once through intraperitoneal at the fifteenth days. Two weeks after the last administration, the animals were weighed, blood samples were collected to obtain serum, and then the animals were killed. The intraperitoneal fluid was collected. The spleen was excised from the animal and weighed immediately and placed in cold PBS-penicillin-streptomycin. The relative was calculated according to the following formula: spleen index (mg/g) = (weight of spleen/body weight).

### 2.6. Phagocytic Activity Assay

The mice were injected intraperitoneally with 0.2 mL of *S. aureus* suspension. One hour later, the mice were killed by ketamine anesthesia, and 3 mL of 3% EDTA was used as an anticoagulant. Then, the intraperitoneal fluid was collected. Intraperitoneal suspension was smeared on glass slides and air-dried. The smear was fixed using methanol for 15 minutes and stained with Giemsa solution for 20 mins. Phagocytic activity was determined by counting the number of phagocytes in a population of 100 phagocytes.

### 2.7. Serum Cytokine Assay

Whole blood was collected and centrifuged at 3000 rpm and 4°C for 10 min, while the upper layer contained the serum. The levels of IFN-*γ*, TNF-*α*, IL-17, and IL-23 in the serum were analyzed by commercial enzyme-linked immunosorbent assay (ELISA) kits (BioLegend, Massachusetts, USA) according to the manufacturer's protocol. The absorbance was measured using ELISA reader at 450 nm.

### 2.8. Splenocytes Isolation

The spleens were gently homogenized and passed through a sterilized copper sieve (200-mesh) to obtain single cell suspensions. Splenocytes suspension was centrifuged at 1000 rpm for 5 minutes. Pellet containing red blood cells was resuspended in tris-buffered NH4Cl pH 7.2 and centrifuged at 1000 rpm for 5 minutes until white pellet was obtained. Splenocytes were washed with 5 ml of PBS-100 unit/ml penicillin-100 *µ*g/ml streptomycin and resuspended in RPMI 1640-FBS 10% medium. Then, splenocytes were used in splenocytes proliferation assay and natural killer cell activity assay.

### 2.9. Splenocytes Proliferation Assay

Cell numbers of splenocytes were counted by haemocytometer. 195 *µ*l of splenocytes (3 × 105 cells/well) and 5 *µ*l of LPS (200 *µ*g/ml) were seeded in 96-well plates. After incubation at 37°C in an incubator with 5% CO_2_ for 48 hours, MTT assay was used. The absorbance was measured at 560 nm. Splenocytes proliferation (%) = ((OD value of okra-treated cells)/(OD value of control)) × 100.

### 2.10. Natural Killer Cell Activity Assay

The splenocytes as the effector (3 × 105 cells/well) was added to human hepatocyte cell line as the target cells (6 × 103 cells/well) to give E/T ratio 50 : 1. They were cultured in 96-well plate and incubated at 37°C in an incubator with 5% CO_2_ for 48 hours. The activity of NK cell was evaluated by the MTT assay. The absorbance was measured at 560 nm. NK cell activity (%) = ((OD value of okra-treated cells)/(OD value of control)) × 100.

### 2.11. Statistical Analysis

Statistical analysis was performed by one-way analysis of variance (ANOVA) followed by Duncan's post hoc test. All analysis was performed using IBM SPSS Statistics 24 software. The results were reported as the mean ± standard deviation (SD) of five repeats. *P* value of <0.05 was considered statistically significant.

## 3. Results

### 3.1. Determination of Polysaccharide Content in Okra Pods

Using the polysaccharide standard regression equation, the polysaccharides content in the stock solution with dose of 100 mg/kg BW was 22.87 mg/mL.

### 3.2. Phagocytic Activity

**Figure 1:**
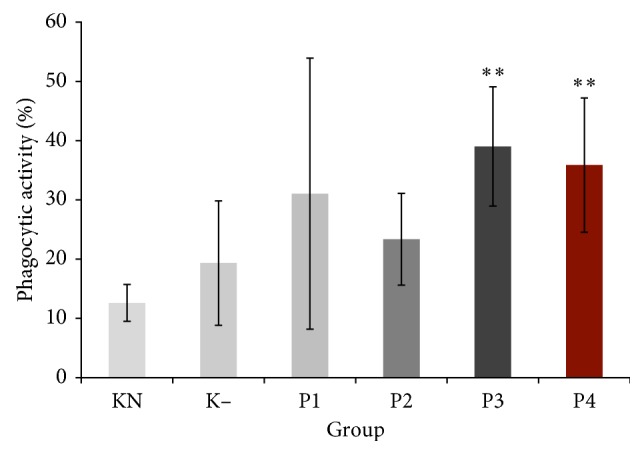
Effect of crude polysaccharide from okra pods on phagocytic activity (%). KN: normal control; K−: negative control; P1, P2, P3, and P4 were treated with 25, 50, 75, and 100 mg/kg BW crude okra polysaccharides, respectively. Each bar represents means ± SD (*n =* 5). ^*∗∗*^*P* < 0.05 compared to normal control.

Phagocytic activity was significantly increased in P3 group and P4 group compared to normal control group and negative control group (*P* < 0.05). The highest increase on phagocytic activity was shown by P3 group. Meanwhile, P1 group and P2 group increased phagocytic activity but did not show significant difference compared with normal and negative control groups (Figure 1).

### 3.3. Cytokines Production

Serum levels of TNF-*α* were significantly increased in P1 group (423.20 ± 128.66 pg/ml), P2 group (460.40 ± 79.28 pg/ml), and P4 group (282.40 ± 80.38 pg/ml) compared to normal control and negative control groups (*P* < 0.05). P3 group (175.50 ± 79.76) also showed slight increase of TNF-*α* level compared to normal control and negative control groups (*P* > 0.05) (Table 1).

In contrast, IL-17 level in P2, P3, and P4 groups was significantly lower than normal control and negative control groups (*P* < 0.05). Although the difference was not significance, P1 showed decrease level of IL-17. There was slight increase in the serum level of IFN-*γ* in P4 group (174.60 ± 64.55 pg/ml) compared to normal control and negative control groups but did not show a significant difference. Meanwhile, there was no difference in the result of IL-23 (Table 1).

### 3.4. Spleen Index

**Figure 2:**
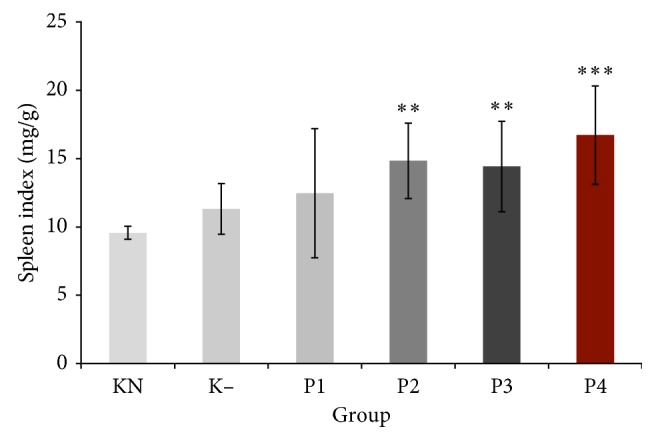
Effect of crude polysaccharide from okra pods on spleen index (mg/g). KN: normal control; K−: negative control; P1, P2, P3, and P4 were treated with 25, 50, 75, and 100 mg/kg BW crude okra polysaccharide, respectively. Each bar represents mean ± SD (*n*=5). ^*∗∗*^*P* < 0.05 compared with normal control. ^*∗∗∗*^*P* < 0.05 compared with normal and negative control groups.

Spleen index was significantly increased in P2 group (14.84 ± 2.76%) and P3 group (14.43 ± 3.31%) compared to normal control group (*P* < 0.05). P4 group at a dose of 100 mg/kg showed highest spleen index (16.72 ± 3.60%) and increased spleen index significantly compared to normal control and negative control groups (*P* < 0.05) (Figure 2).

### 3.5. Splenocytes Proliferation

**Figure 3:**
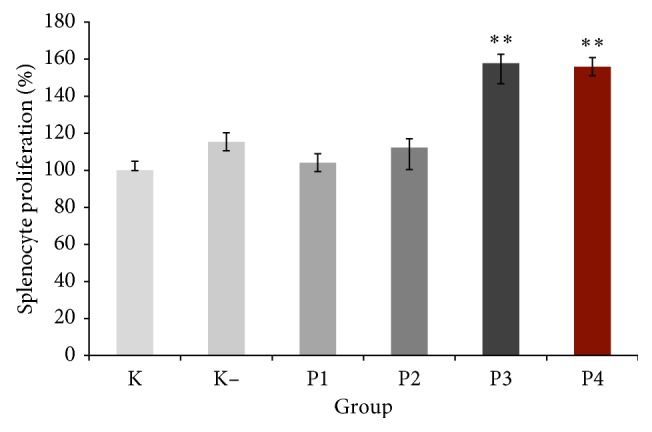
Effect of crude polysaccharide from okra pods on splenocytes proliferation (%). KN: normal control; K−: negative control; P1, P2, P3, and P4 were treated with 25, 50, 75, and 100 mg/kg BW crude okra polysaccharide, respectively. Each bar represents mean ± SD (*n*=5). ^*∗∗*^*P* < 0.05 compared with KN, K−, P1, and P2 groups.

Splenocytes proliferation was significantly increased in the P3 group and P4 group compared with other groups (*P* < 0.05). The highest increase of splenocytes proliferation was demonstrated in P3 group with 157.77 ± 11.06% (Figure 3).

### 3.6. Natural Killer Cell Activity

**Figure 4:**
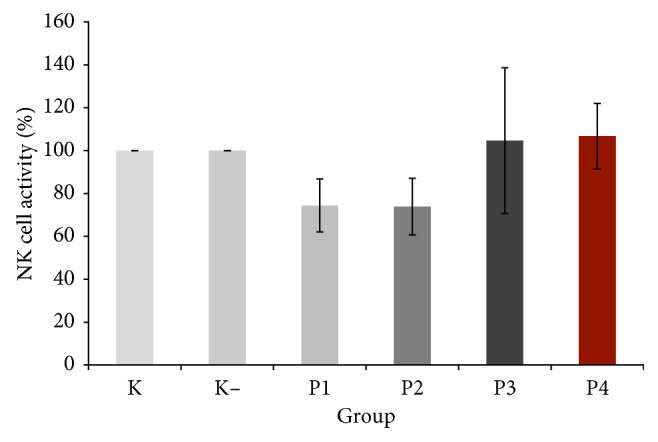
Effect of crude polysaccharide from okra pods on NK cell activity (%). KN: normal control; K−: negative control; P1, P2, P3, and P4 were treated with 25, 50, 75, and 100 mg/kg BW crude okra polysaccharide, respectively. Each bar represents mean ± SD (*n*=5).

K− group and all the treatment groups (P1, P2, P3, and P4) did not show significant increase of natural killer cell activity compared with normal control group. P3 and P4 groups showed slight increase in NK cell activity (104.67 ± 15.32% and 106.75 ± 15.32%) compared to normal control, which was similar to the results of the splenocytes proliferation (Figure 4).

## 4. Discussion

Immune system gives protection to organism against bacterial infection through layered defense, nonspecific immunity, and specific immunity. When bacteria pass through nonspecific defense, the body forms a more complex immune system [10]. These days, the use of immunomodulators to improve immune responses has been considered one of the promising alternatives to prevent bacterial infection [11]. One of the potential compounds as immunomodulator is polysaccharide. In this study, we used crude polysaccharides which contain 22.87 mg/mL of polysaccharides after phenolic sulphuric acid assay was performed.

Nonspecific immunity component that firstly recognizes bacteria is macrophage [12]. Activation of macrophages plays a key role in nonspecific immunity for developing a defensive reaction against pathogens via phagocytosis process [13]. Macrophages release products such as oxygen radicals and tumor necrosis factor that are harmful to bacteria [14].

Polysaccharides regulate the host immune system by activating immune cell-related to lymphocytes, macrophages, and NK cells [15]. The present result demonstrated that crude polysaccharides from okra pods at doses of 75 mg/kg and 100 mg/kg significantly increased phagocytic activity of intraperitoneal phagocytes. Previous observations also demonstrated phagocytes activation by polysaccharides from okra pods both in vitro and in vivo [3, 7]. It has been reported that crude okra polysaccharides significantly increased NO production on RAW264.7 macrophage [7]. In this study, the rise of active phagocytes may lead to increase of proinflammation cytokines production such as TNF-*α*, IFN-*γ*, IL-17, and IL-23 and improves host defense against bacteria. TNF-*α* and IFN-*γ* are cytokines produced by macrophage and Th cell due to all kinds of antigen infections. Macrophage also produced IL-23 to induce activation of Th17. Meanwhile, IL-17 is produced by Th17 specifically. *Staphylococcus aureus* is extracellular bacteria. Extracellular bacteria specifically induce Th naïve to differentiate as Th17 [12]. Based on this consideration, we examine level of TNF-*α*, IFN-*γ*, IL-17, and IL-23.

Cytokines are small proteins produced by cells such as T helper, NK cells, and macrophages that regulate the immune response and inflammation [16]. *S. aureus* presented by active macrophage induces activation of Th-17. T helper-17 produced cytokine such as IL-17, IL-22, and TNF-*α* [12]. Among the cytokines, TNF-*α* is one of the most important proinflammatory cytokines against microbe. The present result showed that serum level of TNF-*α* significantly increased in groups with administration of crude okra polysaccharide at doses of 25, 50, and 100 mg/kg BW. The previous study also reported that crude okra polysaccharides significantly increased TNF-*α* level in RAW264.7 macrophage [7].

From the previous study, okra polysaccharide-induced cytokine production from macrophages through activation of the transcription factor NFкB [17]. The transcription factor NFкB exhibits a potent activity in modulating gene transcription involving TNF-*α*. This present study of TNF-*α* demonstrated that administration of crude okra polysaccharides at a dose of 75 mg/kg did not significantly increase the level of TNF-*α*.

Contrast with this result, the highest increase of phagocytic activity was found in the group with at a dose of 75 mg/kg BW. There is no relation between active macrophages that undergo phagocytosis with production of TNF-*α* level in this study. This result indicated that TNF-*α* was not dominantly produced by macrophages. Crude polysaccharides from okra pods may induce other cells to produce TNF-*α*. Zhou et al. [18] reported that crude extract of *T. wilfordii* strongly inhibits TNF-*α* and IL-1 production. The overproduction of TNF-*α* is related with development of various diseases [19]. Thus, we found this result beneficial because TNF-*α* is suppressed in higher doses. Insignificant level of TNF-*α* in P3 with dose of 75 mg/kg BW could be due to its optimal doses. Based on this result, optimal doses of polysaccharides to increase TNF-*α* were lower doses (25 and 50 mg/kg BW). Although P4 showed significant result, the level of TNF-*α* did not raise as high as P1 and P2 groups.

This study also showed increase of serum level of IFN-*γ* but the difference was insignificant. Meanwhile, serum level of IL-23 did not show any difference. Contrast with serum level of TNF-*α*, all of the treatment groups had decreased serum level of IL-17 compared with normal control group and negative control group. IL-17 is one of the proinflammatory cytokines [10]. These results suggested that crude polysaccharides from okra pods could modulate immune function through promoting and inhibiting cytokines level which help in killing of microbes and control proinflammation cytokine level. In this study, crude polysaccharides from okra pods increased the serum level of IFN-*γ* and controlled serum level of IL-17 and IL-23 possibly to prevent healthy cells from becoming damaged. Both IFN-*γ* and IL-17 are proinflammatory cytokines, and overexpression of proinflammatory cytokines will induce excessive inflammation.

Lymphocytes circulated in the blood and the lymph are also found in large numbers in lymphoid tissues or lymphoid organs. The spleen is a secondary lymphoid organ, a place for maintaining mature naive lymphocytes [20]. Spleen weight and spleen index are changed in response to the nonspecific immunity. It has been reported that immunomodulator can enhance spleen index [21]. This result showed that crude polysaccharides from okra pods at doses of 50, 75, and 100 mg/kg significantly increased the spleen index. This result demonstrated that polysaccharides from okra pods stimulate the immune system by inducing proliferation of immune cells. Therefore, we investigated the effect of polysaccharides from okra pods on the splenocytes proliferation.

Splenocytes are immune cells in the spleen. Splenocytes consist of T cell, B cell, macrophages, and dendritic cells [3]. This result showed that splenocytes proliferation was significantly increased in groups treated with polysaccharides from okra pods at doses of 75 and 100 mg/kg. The previous study also demonstrated an increase of splenocytes proliferation by administration of polysaccharides from *Dendrobium huoshanense* [22]. Recent studies indicated that crude polysaccharides with higher doses increase splenocyte proliferation. Splenocyte proliferation response is related to improved T- or B-lymphocyte immunity which could be an indicator of immune activation [23]. As we report in this study that crude polysaccharides from okra pods induced spleen index, increase of spleen index may occur due to rising of splenocytes proliferation.

As a member of lymphocyte class, NK cells are best known for their nonspecific killing of tumor cells, and there is evidence for their role in controlling infection in the earliest phases of the body's immune response [24]. They can react against and destroy target cells without the help of either major histocompatibility complex- (MHC-)dependent-recognition or prior sensitization, but by exocytosis of perforin-containing granules [25].

Therefore, an NK cell activity assay is a routine method for analysis of a patient's cellular immune response in vitro and can also be used to test the antitumor activities of possible drugs [26]. In this study, we further investigated NK cells activity using human hepatocytes cell line. Our result showed that NK cells activity was able to be restored like normal control group but the difference was not statistically significant. Possibly, polysaccharides target macrophages rather than NK cells.

Immunomodulatory activities of polysaccharides may be due to direct or indirect interaction with immune system components. Complement proteins and monocytes, macrophages, dendritic cells, neutrophils, and lymphocytes have been reported as target responding to polysaccharides [27–29]. Binding of polysaccharides to specific recognition receptors on immune cells trigger diverse signaling pathway and responses [30].

The results of the present study all together showed that crude polysaccharides of the okra pods had the potential to enhance the immune response of some immune components. Treatment groups with higher doses of crude okra polysaccharides increased phagocytic activity, spleen index, and splenocytes proliferation. Meanwhile, treatment groups with lower doses of crude polysaccharides from okra pods showed the highest significant rising of TNF-*α* level. Contrast with other results, treatment groups with higher doses of crude okra polysaccharides showed decrease level of IL-17 as response to prevent overexpression of proinflammatory cytokines.

Based on this study, crude okra polysaccharides could act as an immunomodulator. Crude okra polysaccharides had both immunostimulator activity and immunosuppression activity. Most of the immune components examined in this study showed significant increase and decrease at the doses of 75–100 mg/kg. Crude okra polysaccharide could enhance immune response, showed with rising of phagocytic activity, spleen index, splenocytes proliferation, and TNF-*α* level. Crude okra polysaccharides could suppress immune response (immunosuppression), showed with decrease of IL-17 level.

## 5. Conclusions

We concluded that crude polysaccharides from okra pods could enhance phagocytic activity, spleen index, splenocyte proliferation, and TNF-*α* level, but decrease IL-17 level as a response to prevent overexpression of proinflammatory cytokines. This study suggests that crude polysaccharides from okra pods grown in Indonesia could act as an effective compound to improve immune response.

## Figures and Tables

**Table 1:** Effect of crude polysaccharide from okra pods on cytokines production (pg/ml).

Groups	Cytokine (pg/mL)			
	TNF-*α*	IFN-*γ*	IL-17	IL-23
KN	116.70 ± 78.23	113.00 ± 39.78	208.50 ± 85.67	13.348 ± 0.11
K−	157.59 ± 41.95	156.80 ± 51.00	204.50 ± 84.65	13.300 ± 0.01
P1	423.20 ± 128.66^*∗∗∗*^	98.40 ± 50.98	127.50 ± 78.77	13.377 ± 0.04
P2	460.40 ± 79.28^*∗∗∗*^	139.20 ± 23.38	73.25 ± 22.53^*∗∗*^	13.319 ± 0.03
P3	175.50 ± 79.76	97.20 ± 41.44	78.25 ± 50.42^*∗∗*^	13.264 ± 0.10
P4	282.40 ± 80.38^*∗∗*^	174.60 ± 64.55	48.25 ± 16.62^*∗∗*^	13.298 ± 0.08

KN: normal control; K−: negative control; P1, P2, P3, and P4 were treated with 25, 50, 75, and 100 mg/kg BW crude okra polysaccharide, respectively. Values are represented as mean ± SD (*n* = 5). ^*∗∗*^*P* < 0.05 compared with KN group. ^*∗∗∗*^*P* < 0.05 compared with KN, K−, P3, and P4 groups; TNF: tumor necrosis factor; IFN: interferon; IL: interleukin.

## Data Availability

The data used to support the findings of this study are included within the article.
